# Variability of high-sensitivity cardiac troponin T and I in asymptomatic patients receiving hemodialysis

**DOI:** 10.1038/s41598-021-96658-0

**Published:** 2021-08-30

**Authors:** Wanwarang Wongcharoen, Teetad Chombandit, Arintaya Phrommintikul, Kajohnsak Noppakun

**Affiliations:** 1 Division of Cardiology, Department of Internal Medicine, Faculty of Medicine, Chiang Mai University, Chiang Mai, Thailand; 2 Division of Nephrology, Department of Internal Medicine, Faculty of Medicine, Chiang Mai University, Chiang Mai, Thailand; 3 Pharmacoepidemiology and Statistics Research Center (PESRC), Faculty of Pharmacy, Chiang Mai University, Chiang Mai, Thailand

**Keywords:** Cardiology, Medical research, Nephrology

## Abstract

Variation of high-sensitivity cardiac troponin I and T (hs-cTn) during hemodialysis has been observed. Observational studies demonstrated the increased incidence of adverse cardiovascular events after long compared to short interdialytic intervals. Therefore, we aimed to compare variation of hs-cTnI and hs-cTnT before and after hemodialysis and between short and long interdialytic intervals. We enrolled 200 asymptomatic patients receiving regular hemodialysis. The hs-cTnI and hs-cTnT levels were measured before and after hemodialysis on the day after short and long interdialytic intervals. Mean age was 62.3 ± 14.8 years (Male 55.5%). Prevalence of increased hs-cTnI and hs-cTnT was 34.5% and 99.0%, respectively. The median ± interquartile range of hs-cTnT increased significantly after hemodialysis during short and long interdialytic intervals. However, hs-cTnI level did not increase after hemodialysis during short and long intervals. We found that levels of hs-cTnI and T did not differ between short interdialytic and long interdialytic intervals. We demonstrated higher prevalence of elevated hs-cTnT in patients with regular hemodialysis compared to hs-cTnI. The rise of hs-cTnT was observed immediately after hemodialysis but no significant change of hs-cTnI was noted. Accordingly, hs-cTnI may be preferable as a diagnostic marker in patients with suspected acute myocardial infarction than hs-cTnT.

## Introduction

The prevalence of end-stage renal disease (ESRD) and incidence of hemodialysis has distinctly increased during the past two decades^1^. Coronary artery disease, especially acute coronary syndrome, is a major cause of cardiac hospitalizations and cardiac deaths in hemodialysis population^2^. According to recent guideline recommendation, high-sensitivity cardiac troponin (hs-cTn) is recommended for the diagnosis of acute myocardial infarction^3,4^. It is defined as the rise of hs-cTn more than the 99th percentile of the upper reference limit (URL) or a rise of hs-cTn more than 20% if baseline level is elevated^4^. However, the cutoff level of hs-cTn recommended by international guidelines was derived mostly from patients without chronic kidney disease. Remarkably, it has been well described that the baseline hs-cTn levels in asymptomatic hemodialysis patients were higher than general population^5^. Recent studies have shown that majority of patients with ESRD with chronic hemodialysis had baseline hs-cTn above the 99th percentile URL^6,7^. Furthermore, the variation of hs-cTn level before, during, and after hemodialysis has been observed^8–10^. With this regard, the diagnosis of acute coronary syndrome in patients undergoing regular hemodialysis is challenging.

The hs-cTn I and hs-cTn T are considered the gold-standard biomarkers for detection of myocardial injury^4^. However, these two biomarkers have different biochemical characteristics and use different cut-off values^11^. In addition, previous studies have demonstrated the conflicting findings between the results of cTn I and cTn T in some population^12,13^. Notably, a greater number of patients having an increased cTn T compared to cTn I has been reported in chronic hemodialysis patients^7^. Currently, the use of conventional cTn has been replaced by hs-cTn due to the much higher sensitivity of the latter^4^. Nevertheless, the prevalence of elevated hs-cTn I and hs-cTn T levels in hemodialysis patients has rarely been explored. Therefore, we conducted this study to compare the prevalence of increased hs-cTn I and hs-cTn T in hemodialysis patient. The alteration of hs-cTn I and hs-cTn T pre-dialysis and post-hemodialysis in asymptomatic patients was also investigated. Moreover, observational studies have shown that the incidence of adverse cardiovascular events significantly increased during the period after long interdialytic interval compared to short interdialytic interval^14,15^. Accordingly, we also sought to examine the difference of hs-cTn I and hs-cTn T levels between long and short interdialytic interval.

## Methods

We enrolled asymptomatic patients diagnosed with ESRD, aged > 18 years, who had undergone regular hemodialysis (two or three times a week) for more than 90 days in the study. We excluded patients who had a recent diagnosis of acute myocardial infarction, heart failure and pulmonary embolism within previous 6 months, had major surgery and trauma within previous 4 weeks, and had coronary and/or valvular intervention within previous 6 months. The hs-cTn I and T levels were measured in all subjects before hemodialysis and after hemodialysis session on the day after short interdialytic interval and the day after long interdialytic interval. With this regard, each patient had four levels of hs-cTn I and four levels of hs-cTn T. The hs-cTn T values were evaluated with electrochemiluminescence immunoassay by using the Cobas e801 system (Roche Diagnostics). The detection limit of hs-cTn T was 3 ng/L, a cut-off point at 99th percentile was 14 ng/L, and a coefficient of variation of less than 10% was at 13 ng/L. The hs-cTn I values were evaluated with chemiluminescence microparticle immunoassay (CMIA) by using the ARCHITECT i2000SR system (Abbott Diagnostics). The detection limit of hs-cTn I was 3.2 ng/L, a cut-off point at 99th percentile was 26.2 ng/L, and a 10% coefficient of variation was 4.7 ng/L.

We defined the short and long interdialytic intervals as follows. For the patients who have been receiving thrice weekly hemodialysis, the long interdialytic interval was 2-day interval between hemodialysis sessions. The short interdialytic interval was 1-day interval between hemodialysis sessions. For those who have been received twice weekly hemodialysis, the long interdialytic interval was 3-day interval between hemodialysis sessions. The short interdialytic interval was 2-day interval between hemodialysis sessions.

Clinical data were recorded, including age, gender, duration of hemodialysis, medications, echocardiographic results within 1 year, ultrafiltration volume, comorbidity, and blood chemistry. This study was approved by the institutional research board of Faculty of Medicine, Chiang Mai University (Approval No. 112/2563). The study procedure was performed according to the Declaration of Helsinki. Informed consent was obtained from all participants.

### Statistical analysis

Results are expressed as mean ± SD, unless otherwise specified and compared between group with t-test or paired t-test. Results with non-normal distribution are expressed as median (interquartile range) with non-parametric test. The numerical variables were compared within groups with paired t test or Wilcoxon matched paired sign-rank test. Mann–Whitney U test as appropriate. Proportions were compared by Fisher’s exact test. Univariate and multivariate linear regression analysis was used to examine the association between potential variables and the change of hs-cTn after hemodialysis. *p* values < 0.05 was considered statistically significant. Statistical software package IBM SPSS Statistics for Windows, version 23 (IBM Corp., Armonk, NY, USA, https://www.ibm.com/products/spss-statistics) was used for analysis.

### Ethics approval and consent to participate

The Effect of long and short interdialytic interval of chronic hemodialysis on heart rate variability in patients with end-stage renal disease was approved by the ethics committee of the Faculty of Medicine, Chiang Mai University, approval number 112/2563. The investigations were carried out in accordance with the Declaration of Helsinki, including written informed consent of all participants.

## Results

Baseline characteristics of the studied population are shown in Table 1. The mean age was 62.3 ± 14.8 years. Male was prevalent in 55.5%. The prevalence of hypertension, diabetes mellitus, and coronary artery disease was 91.0%, 45.0% and 13.0%, respectively. There were 189 (94.5%) patients receiving hemodialysis thrice a week. The rest were hemodialyzed twice a week.

**Table 1:** Basic characteristics of the studied population.

Characteristic	Value (N = 200)
Age (year)	62.3 ± 14.8
Male *Smoking status* - No smoking - Ex-smoker - Current smoker	111 (55.5%) 146 (73.0%) 41 (20.5%) 4 (2.0%)
*Frequency of hemodialysis* - Thrice a week - Twice a week	189 (94.5%) 11 (5.5%)
*Vascular access* - Arteriovenous fistula - Permanent catheter - Arteriovenous graft	139 (69.5%) 51 (25.5%) 8 (4.0%)
*Underlying disease* - Hypertension - Hyperlipidemia - Diabetes mellitus - Coronary artery disease - Atrial fibrillation - Cerebrovascular disease - Peripheral artery disease	182 (91.0%) 107 (53.5%) 90 (45.0%) 26 (13.0%) 23 (11.5%) 21 (10.5%) 7 (3.5%)
*Medications* - Calcium channel blocker - Beta-blocker - Statin - Diuretics - Anti-platelets - Alpha adrenergic blocker - ACEI or ARB - Warfarin	129 (64.5%) 127 (63.5%) 114 (57.0%) 92 (46.0%) 72 (36.0%) 63 (31.5%) 51 (25.5%) 18 (9.0%)
*Laboratory* - Sodium (mmol/L) - Potassium (mmol/L) - Albumin (g/dl) - Hemoglobin (g/dl)	136.7 ± 3.3 4.4 ± 0.7 4.0 ± 0.4 10.4 ± 1.4

ACEI = angiotensin converting enzyme inhibitor; ARB = angiotensin receptor blocker.

The mean body weight prior to hemodialysis was greater in long interdialytic interval compared to short interdialytic interval (62.8 ± 16.7 kg vs. 62.3 ± 16.8 kg, P < 0.001). Likewise, the mean net ultrafiltration volume and the mean ultrafiltration rate was significantly greater in long interdialytic interval compared to short interdialytic interval. Table 2 shows hemodialysis parameters between short and long interdialytic intervals.

**Table 2:** Hemodialysis parameters between short and long interdialytic intervals.

Parameters	Short interdialysis interval	Long interdialysis interval	*p* value
Body weight (kg) - Pre-dialysis - Post-dialysis	62.3 ± 16.8 60.2 ± 16.3	62.8 ± 16.7 60.5 ± 16.3	< 0.001 < 0.001
Blood pressure (mmHg) - SBP pre-dialysis - DBP pre-dialysis - SBP post-dialysis - DBP post-dialysis	143.6 ± 20.0 76.5 ± 12.1 137.9 ± 17.1 75.4 ± 11.5	144.3 ± 20.3 76.3 ± 12.3 137.3 ± 16.8 75.7 ± 13.3	0.63 0.84 0.65 0.73
Net filtration volume (ml)	2312.5 ± 948.8	2623.5 ± 963.1	< 0.001
Ultrafiltration rate (ml/hour/kg)	9.45 ± 3.73	10.70 ± 3.93	< 0.001
Hypotension during hemodialysis (%)	5 (2.6%)	4 (2.1%)	0.15

SBP = systolic blood pressure, DBP = diastolic blood pressure.

**Figure 1:**
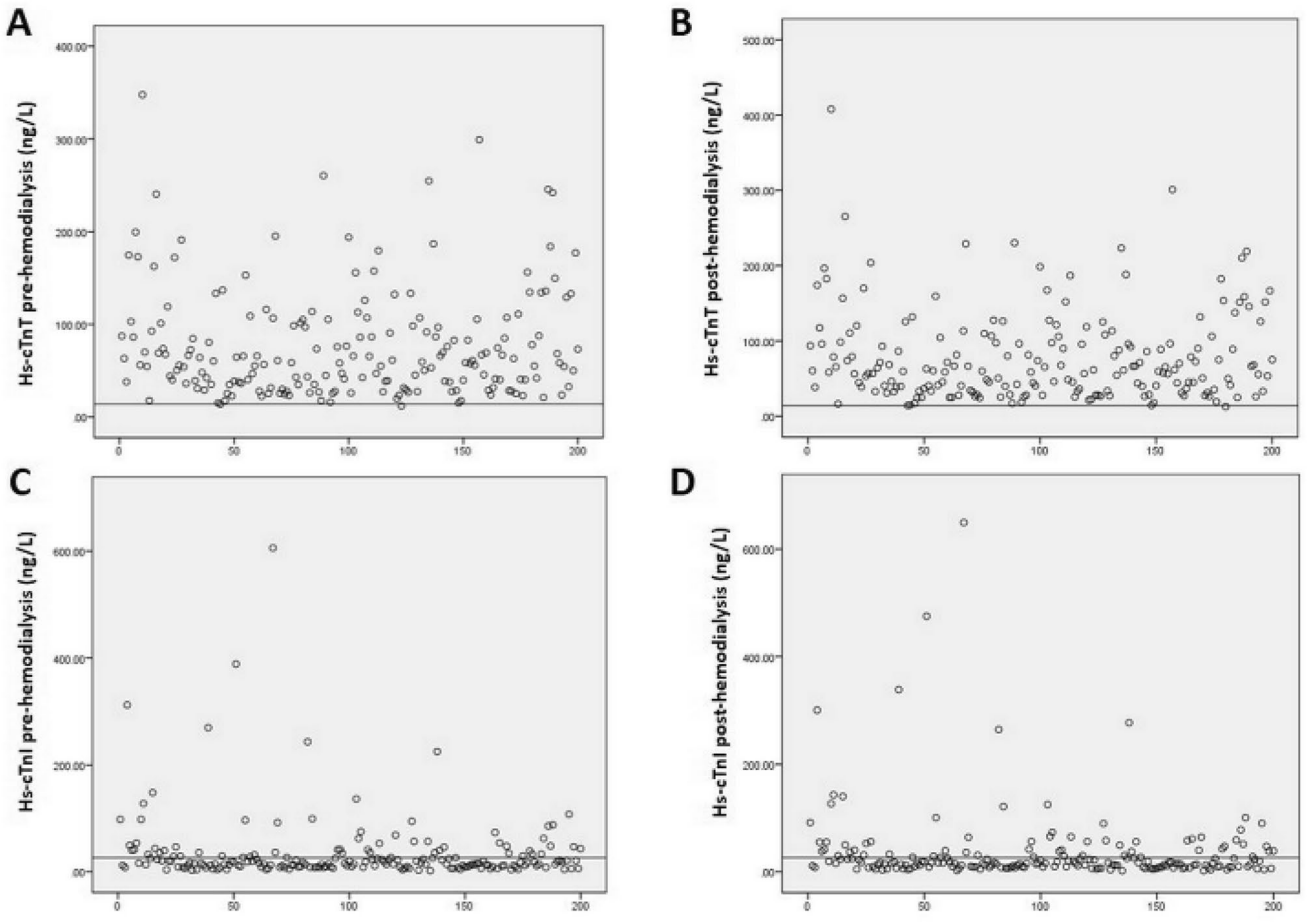
Individual variation of high-sensitivity cardiac troponin T and high-sensitivity cardiac troponin I pre-hemodialysis and post-hemodialysis during short interdialytic interval. (**A**) High-sensitivity cardiac troponin T pre-hemodialysis (line indicates cut-off level of 14 ng/L). (**B**) High-sensitivity cardiac troponin T post-hemodialysis (line indicates cut-off level of 14 ng/L). (**C**) High-sensitivity cardiac troponin I pre-hemodialysis (line indicates cut-off level of 26.2 ng/L). (**D**) High-sensitivity cardiac troponin I post-hemodialysis (line indicates cut-off level of 26.2 ng/L).

**Figure 2:**
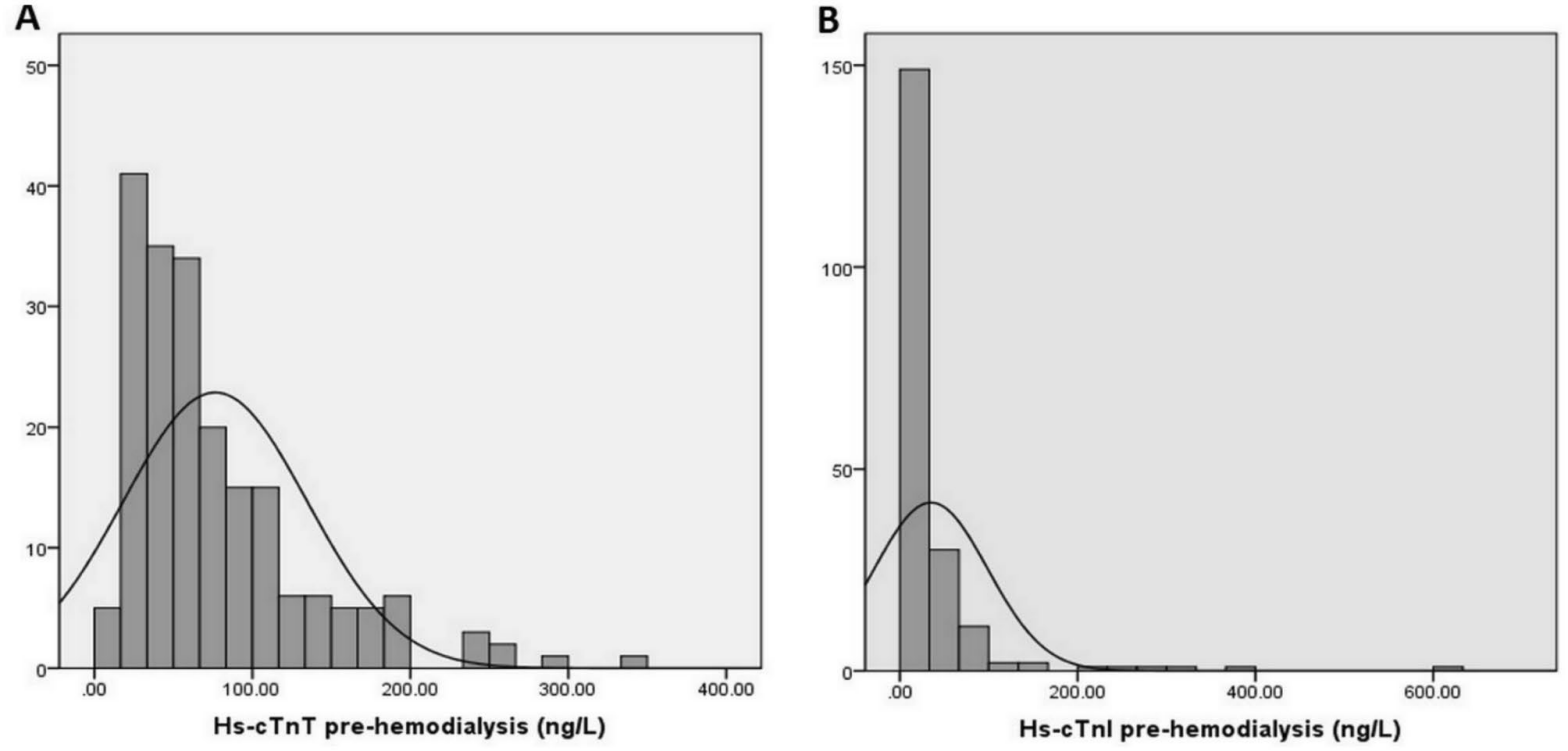
Histogram of high-sensitivity cardiac troponin T (**A**) and I (**B**) levels before hemodialysis.

We demonstrated that 198 (99.0%) patients had an increased level of hs-cTn T above 99th percentile of the URL in both short and long interdialytic intervals. On the other hand, only 64 (32.0%) and 69 (34.5%) patients had increased hs-cTn I during short and long interdialytic intervals, respectively (Fig. 1). The histogram of hs-cTn T and I levels before hemodialysis are presented in Fig. 2. The wider range of hs-cTn T levels among interindividual patients was observed compared to hs-cTn I levels.

Compared pre- and post-hemodialysis, the median level of hs-cTn T increased significantly after hemodialysis, similarly during short interdialytic interval (59.0 ng/L, IQR 35.43–100.45 ng/L vs. 60.55 ng/L, IQR 35.7–98.3 ng/L, *P* < 0.001) and during long interdialytic interval (60.6 ng/L, IQR 36.4–101.2 ng/L vs. 61.7 ng/L, IQR 36.9–108.9 ng/L, *P* < 0.001). In contrast, the level of hs-cTn I did not increase significantly after hemodialysis during short interdialytic interval (17.1 ng/L, IQR 9.8–34.9 ng/L vs. 16.6 ng/L, IQR 9.5–37.4 ng/L, *p* = 0.59) and long interdialytic interval (18.4 ng/L, IQR 9.4–37.0 ng/L vs. 19.4 ng/L, IQR 10.2–33.8 ng/L, *p* = 0.59) (Table 3).

**Table 3:** The change of biomarkers before and after hemodialysis in short and long interdialytic intervals.

Biomarker	Short interdialysis interval		*p* value*	Long interdialysis interval		*p* value*
	Pre-dialysis	Post-dialysis		Pre-dialysis	Post-dialysis	
Hs-cTnI (ng/L)	17.1 (9.8, 34.9)	16.6 (9.5, 37.4)	0.59	18.4 (9.4, 37.0)	19.4 (10.2, 33.8)	0.59
Hs-cTnT (pg/L)	59.0 (35.4, 100.4)	60.5 (35.7, 98.3)	< 0.001	60.6 (36.4, 101.2)	61.7 (36.9, 108.9)	< 0.001

*Compared between pre- and post-hemodialysis, hs-cTn = high-sensitivity cardiac troponin.

Data are presented as median level (interquartile range).

We also compared levels of hs-cTn between short interdialysis and long interdialysis intervals. The levels of hs-cTn I and T did not differ between short interdialytic and long interdialytic intervals, either pre-hemodialysis or post-hemodialysis level (Table 3).

We performed the univariate and multivariate linear regression to examine the association of the change of hs-cTn levels after hemodialysis and potential variables including age, medications, hemoglobin (Hb) level, vascular access, underlying coronary artery disease (CAD) and ultrafiltration rate (Table 4).

**Table 4:** Univariate and Multivariate linear regression analysis showing the association of potentialvariables and the change of hs-cTn after hemodialysis.

					Long interdialytic interval			
	∆ hs-cTn T		∆ hs-cTn I		∆ hs-cTn T		∆ hs-cTn I	
	Univariate β coefficient (95% CI), *p* value	Multivariate β coefficient (95% CI), *p* value	Univariate β coefficient (95% CI), *p* value	Multivariate β coefficient (95% CI), *p* value	Univariate β coefficient (95% CI), *p* value	Multivariate β coefficient (95% CI), *p* value	Univariate β coefficient (95% CI), *p* value	Multivariate β coefficient (95% CI), *p* value
Age (per year)	0.05 (− 0.06, 0.16) *p* = 0.382	**0.14** **(0.01, 0.27)** ***p* = 0.035**	0.07 (− 0.04, 0.17) *p* = 0.218	0.10 (− 0.03, 0.22) *p* = 0.124	0.03 (− 0.07, 0.14) *p* = 0.537	**0.13** **(0.01, 0.24)** ***p* = 0.030**	**0.22** **(0.03, 0.41)** ***p* = 0.026**	0.21 (− 0.01, 0.43) *p* = 0.066
ACEI/ARB	2.23 (− 1.57, 6.02) p = 0.249	2.21 (− 1.72, 6.13) p = 0.269	− 0.23 (− 3.91, 3.46) p = 0.903	− 1.03 (− 4.75, 2.70) p = 0.587	− 0.69 (− 4.38, 2.99) p = 0.711	− 1.82 (− 5.34, 1.70) p = 0.308	− 3.72 (− 10.5, 3.01) p = 0.277	− 4.21 (− 11.12, 2.70) p = 0.231
BB	− 0.83 (− 4.37, 2.72) p = 0.646	− 1.21 (− 4.96, 2.54) p = 0.525	− 0.79 (− 4.21, 2.63) p = 0.650	− 1.37 (− 4.93, 2.18) p = 0.447	2.29 (− 1.12, 5.70) 0.187	1.77 (− 1.60, 5.14) p = 0.301	0.13 (− 6.15, 6.41) p = 0.967	1.75 (− 4.88, 8.38) p = 0.603
Hb level (per 1 g/dL)	− 1.01 (− 2.18, 0.16) *p* = 0.089	− 1.14 (− 2.40, 0.12) *p* = 0.075	− **1.33** **(**− **2.43, **− **0.23)** ***p* = 0.018**	− **1.46** **(**− **2.66, **− **0.27)** ***p* = 0.016**	− 0.10 (− 1.21, 1.02) 0.863	− 0.41 (− 1.53, 0.72) *p* = 0.478	0.19 (− 1.85, 2.23) *p* = 0.855	− 0.25 (− 2.46, 1.96) *p* = 0.824
Vascular access	0.06 (− 3.74, 3.85) *p* = 0.977	0.45 (− 3.49, 4.40) *p* = 0.820	2.84 (− 0.75, 6.43) *p* = 0.121	2.08 (− 1.67, 5.82) *p* = 0.275	1.23 (− 2.42, 4.87) 0.508	1.42 (− 2.11, 4.95) *p* = 0.429	− 2.33 (− 8.92, 4.26) *p* = 0.486	− 5.33 (− 12.28, 1.61) *p* = 0.131
CAD	0.83 (− 4.11, 5.78) *p* = 0.740	− 0.21 (− 5.22, 4.81) *p* = 0.935	4.22 (− 0.45, 8.90) *p* = 0.076	4.07 (− 0.69, 8.83) *p* = 0.093	3.02 (− 1.72, 7.76) *p* = 0.211	4.33 (− 0.18, 8.84) *p* = 0.060	− 3.02 (− 11.61, 5.57) *p* = 0.489	− 3.21 (− 12.07, 5.66) *p* = 0.477
Ultrafiltration rate (per 1 mL/h/kg)	0.23 (− 0.22, 0.68) *p* = 0.312	− 0.38 (− 1.10, 0.33) *p* = 0.293	**0.57** **(0.15, 0.99)** ***p* = 0.009**	**0.89** **(0.22, 1.57)** ***p* = 0.010**	**1.06** **(0.67, 1.44)** ***p* < 0.001**	**0.99** **(0.40, 1.58)** ***p* = 0.010**	0.46 (− 0.28, 1.21) *p* = 0.218	**1.70** **(0.54, 2.86)** ***p* = 0.004**

ACEI/ARB = angiotensin converting enzyme inhibitor/angiotensin receptor blocker, BB = beta-blocker, CAD = coronary artery disease, Hb = Hemoglobin, hs-cTn = high-sensitivity cardiac troponin.

We found that older age was associated with the greater change of hs-cTn T during short and long interdialytic interval. However, age was not associated with the change of hs-cTn I after hemodialysis. In addition, we demonstrated that ultrafiltration rate was an independent factor to predict the change of hs-cTn I during short and long interdialytic interval and the change of hs-cTn T during long interdialytic interval. Furthermore, the lower Hb level was independently associated with the greater change of hs-cTn I during short interdialytic interval.

There were 127 (63.5%) patients receiving beta-blocker and 63 (31.5%) patients receiving angiotensin converting enzyme inhibitor (ACEI) or angiotensin receptor blocker (ARB). Due to the fact that these medications have been shown to protect myocardial ischemia, we explored the effect of these medications on the change of hs-cTn. We demonstrated that the change of hs-cTn T and I was similar in those receiving beta-blocker and those without beta-blocker therapy. Also, patients with and without ACEI/ARB therapy had the comparable change of hs-cTn T and I.

We examined the effect of different vascular access on the change of hs-cTn. We analyzed the change of hs-cTn separately in the 147 (73.5%) patients with arteriovenous (AV) fistula/graft and 53 (26.5%) patients with central venous catheter. We demonstrated the similar change of hs-cTn T and I before and after hemodialysis between two groups.

There were 26 (13%) patients with history of CAD in the studied population. The presence of CAD was not associated with the greater change of hs-cTn after hemodialysis after multivariate analysis (Table 4). Interestingly, we observed that hs-cTn T levels before and after hemodialysis were significantly higher in patients with history of CAD than those without. In contrast, hs-cTn I levels were not different between those with and without CAD. The data is shown in Table 5.

**Table 5:** The difference of biomarkers between patients with and without history of coronary artery disease.

Biomarker	Hs-cTn T (ng/L)		*p* value	Hs-cTn I (ng/L)		*p* value
	No CAD	CAD		No CAD	CAD	
Pre-dialysis during SI	56.1 (IQR 33.8–91.1)	94.4 (IQR 62.3–122.6)	0.008	16.1 (IQR 9.5–32.2)	19.6 (IQR 11.9–43.3)	0.204
Post-dialysis during SI	57.7 (IQR 33.4–95.9)	92.8 (IQR 62.0–121.7)	0.005	16.0 (IQR 9.1–32.0)	20.0 (IQR13.5–43.8)	0.193
Pre-dialysis during LI	56.5 (IQR 35.0–95.0)	98.6 (IQR 68.0–120.4)	0.001	17.0 (IQR 9.3–33.6)	24.7 (IQR 12.3–51.6)	0.076
Post-dialysis during LI	58.4 (IQR 33.8–98.1)	99.9 (IQR 71.6–121.4)	0.001	19.0 (IQR 9.9–33.3)	26.2 (IQR 14.4–46.8)	0.056

CAD = coronary artery disease, hs-cTn = high-sensitivity cardiac troponin, LI = long interdialytic interval, SI = short interdialytic interval.

Data are presented as median level and interquartile range (IQR).

## Discussion

### The elevation of hs-cTn T and I

The hs-cTn level is recommended for the diagnosis of acute myocardial infarction but its cutoff level is derived from epidemiological data in general population without ESRD. Several investigators have shown that patients with regular hemodialysis have elevated hs-cTn levels compared to general population^5–7,16^. This could be partly explained by the occurrence of microinfarction, heart failure, degenerative changes or other myocardial pathology in patients with ESRD^9^. Previous study has demonstrated that greater number of patients with regular hemodialysis had an elevated cTn T level compared to cTn I level^7,17,18^. In similar, the prevalence of increased hs-cTn T was much higher than hs-cTn I in our studied population. The elevation of troponin levels in dialysis patients could be related to other factors beyond the ischemic cause, such as left ventricular systolic and diastolic dysfunction, left ventricular hypertrophy, myocardial stunning, volume overload, microvascular disease, endothelial dysfunction, and decreased renal clearance^18–20^.

The disparity between the prevalence of increased levels of hs-cTn I and T in patients with ESRD may be explained by the fact that they have different biochemical, genetic, kinetic features and have dissimilar analytical performances^11^. The cellular distribution was different between hs-cTn I and T. It has been shown that cTn T has higher tissue concentration and free cytoplasmic concentrations than cTnI^21,22^. As a result, hs-cTn T may release more early with greater amount than hs-cTn I during myocardial injury. This was in line with previous studies showing that cTn T levels increased in some patients with neuromuscular disease or inflammatory myopathy but no rising of cTn I was noted^12,13^.

Due to the fact that almost all patients in our study had elevated level of hs-cTn T, the cut-off value of hs-cTn T used currently for the diagnosis of acute myocardial infarction would be of no benefit in patients with chronic hemodialysis. Therefore, a significant rise and fall of sequential hs-cTn T levels should be used in favor of single elevated level of hs-cTn T to make the diagnosis of acute myocardial infarction. Interestingly, we demonstrated that only one-third of patients with regular hemodialysis had elevated hs-cTn I. Of importance, we demonstrated that patients with history of CAD had significantly higher level of hs-cTnT compared to those without. However, no significant difference of hs-cTn I level was noted between those with and without history of CAD. From a practical standpoint, hs-cTn I level may be a more favorable biomarker than hs-cTn T level in the evaluation of the patients with suspected acute myocardial infarction, particularly in those with history of CAD.

Previous study has explored the prognostic value of hs-cTn T in pre-dialysis advanced chronic kidney disease patients. They found that those with glomerular filtration rate < 20 ml/min/1.73 m^2^ had a 2.5-fold increase in hs-cTn T cutoff level to predict long-term cardiovascular outcome compared to the standard cutoff level used in those with normal renal function. It has been suggested that the cutoff level of hs-cTn T should be 35 ng/L in this group of patient.^23^ Nevertheless, the data regarding prognostic values of cTn in patients with ESRD are conflicting. The cTn T has been shown to have better predictor of long-term cardiovascular outcomes than cTn I in patients with chronic hemodialysis in some studies^7,24–26^. However, conventional cardiac troponin assay was used in most of the studies which may have limitation of sensitivity and specificity as compared to high-sensitivity assay, especially in ESRD patients^7,24,26^. Larger studies using hs-cTn to predict long-term outcome in patients with ESRD should be warranted to clarify this issue.

### The change of hs-cTn T and I after hemodialysis

Interestingly, we demonstrated that hs-cTn T level increased significantly after hemodialysis during both short and long interdialytic intervals. Nevertheless, we did not observe the significant change of hs-cTn I after hemodialysis. Our results were similar to previous studies that showed the increase in cTn T, but not cTn I, in patients after hemodialysis^24,27^. On the contrary, other two studies showed that hs-cTn I and hs-cTn T decreased slightly after hemodialysis^9,10^. However, the number of patients in those two studies was small in which only 10 and 20 patients were included.

It is plausible that hs-cTn T may have higher sensitivity to detect minor myocardial injury during hemodialysis compared to hs-cTn I. The hemodialysis has been shown to reduce myocardial blood flow^28^. In accordance, previous study showed that high ultrafiltration rate was associated with increased hs-cTn levels during hemodialysis^29^. High volume depletion during hemodialysis may cause hemodynamic compromise leading to myocardial ischemia. Nevertheless, our result showed that ultrafiltration rate was associated with the change of both hs-cTn T and I level after hemodialysis. The dissimilar biochemical characteristics between these two biomarkers may also partly explain the different findings. It has been described that hs-cTn I can adsorb onto the dialysis membrane because of its hydrophobicity which may result in the lack of hs-cTn I elevation after hemodialysis^11^.

### The change of hs-cTn during short and long interdialytic intervals

Several observational studies have shown that the incidence of adverse cardiovascular events significantly increased during the period after long interdialytic interval compared to that after short interdialytic interval^14,15^. Numerous factors have been reported to contribute to the major adverse cardiac events during long interdialytic interval compared to short interdialytic interval. Greater degree of hypervolemic status during long interdialytic interval may induce structural and functional disorders in myocardium, leading to the occurrence of myocardial damage. Electrolyte imbalance and the disorder in autonomic nervous system during long interdialytic interval may result in the increased risk of cardiac arrhythmias. In addition, the increase in oxidative stress, inflammation and abnormal calcium or phosphate metabolism during long interdialytic interval, may play some roles in atherosclerosis of coronary artery^30–33^. Of interest, we did not find any difference in hs-cTn I and T levels between short and long interdialytic intervals. Our results suggest that myocardial injury may not be a major factor contributing to worse prognosis during long interdialytic interval.

Our study has several limitations. First, the fluid overload and fluid management are significant confounding factors affecting cardiac ischemia via stretching and shrinking cycles. However, we did not assess the fluid status of the patients by any bioimpedance measurements or cardiac biomarkers such as b-type natriuretic peptide (BNP) or N-terminal pro BNP (NTproBNP). We examined only the ultrafiltration volume, the ultrafiltration rate and body weight change which may not be insufficient to evaluate the fluid status of the patients. Second, we did not assess the inflammatory marker in our studied population. This issue should be explored in future studies. Third, we did not include patients with non-dialysis ESRD and those with chronic peritoneal dialysis. Therefore, our findings could not be applied in these population. Future studies are warrant to explore the difference of hs-cTn variability between our studied population and those with peritoneal dialysis and non-dialysis ESRD patients.

## Conclusion

We demonstrated the higher prevalence of elevated hs-cTn T in patients with regular hemodialysis compared to hs-cTn I. In addition, the rise of hs-cTn T was observed immediately after hemodialysis but no change of hs-cTn I was noted. Nevertheless, the levels of both hs-cTn I and T did not change between short and long interdialytic intervals. Our results indicate that hs-cTn T may be more sensitive than hs-cTn I to detect minor degree of myocardial injury in patients receiving hemodialysis. However, from a practical standpoint, hs-cTn I may be more favorable as a diagnostic marker in patients with suspected acute myocardial infarction than hs-cTn T.

## Data Availability

The informed consent given by effect of long and short interdialytic interval of chronic hemodialysis on heart rate variability in patients with end-stage renal disease study participants does not cover data posting in public databases. However, data are available upon request should be sent to kajohnsak.noppakun@cmu.ac.th and are subject to approval by the Faculty of Medicine, Chiang Mai University Ethics Committee.
